# Supplementary figures and images for: The auditory brainstem response to natural speech is not affected by selective attention
Source: PLoS Biol. 2025 Oct 6;23(10):e3003407. doi: 10.1371/journal.pbio.3003407 (PMC12500158; doi:10.1371/journal.pbio.3003407)

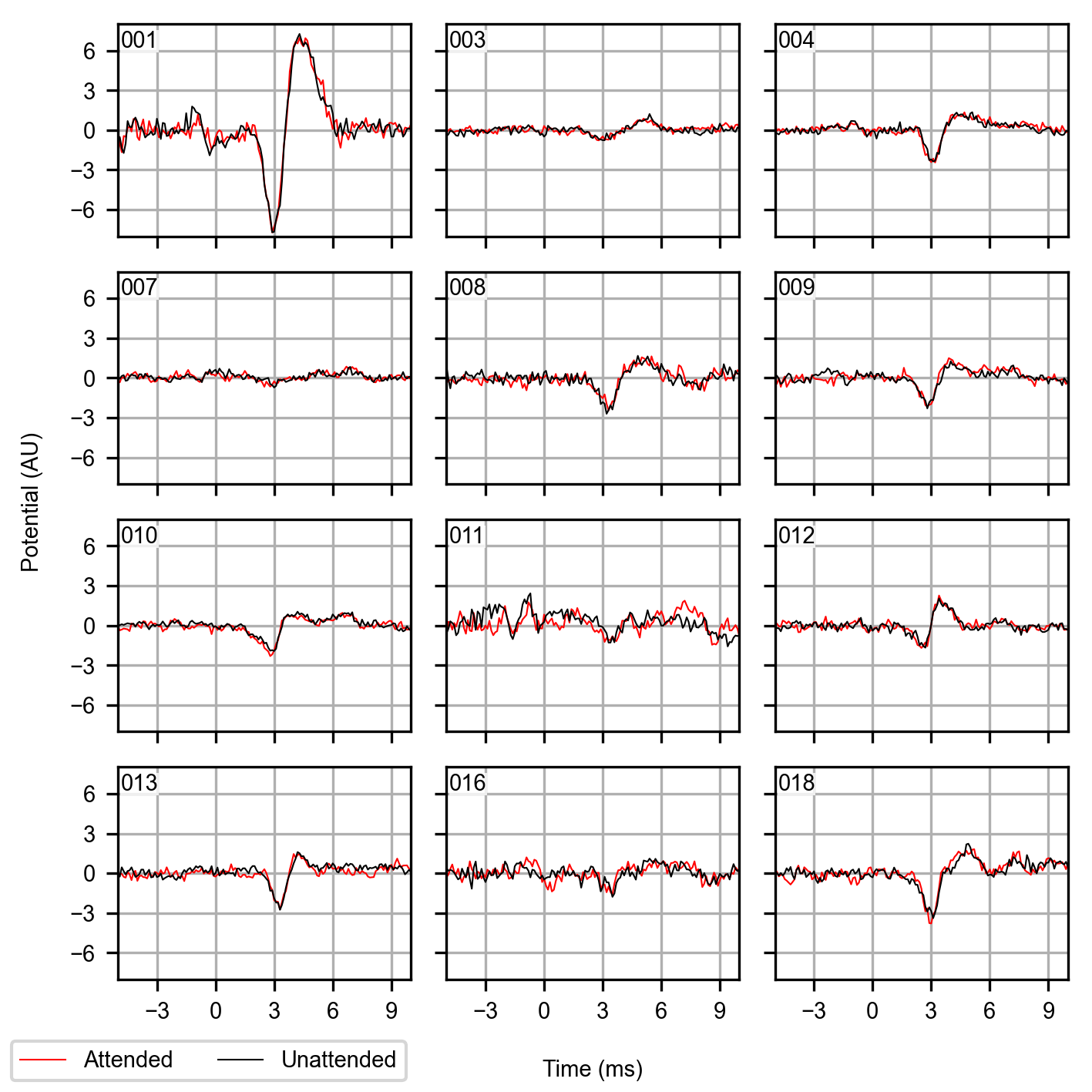

Supplement: S1 Fig — The CAPs to attended (red) and unattended (black) speech are shown for all subjects for whom we obtained a response with the eardrum electrode. Grand averages shown in Fig 1a. (TIF) [file pbio.3003407.s001.tif]

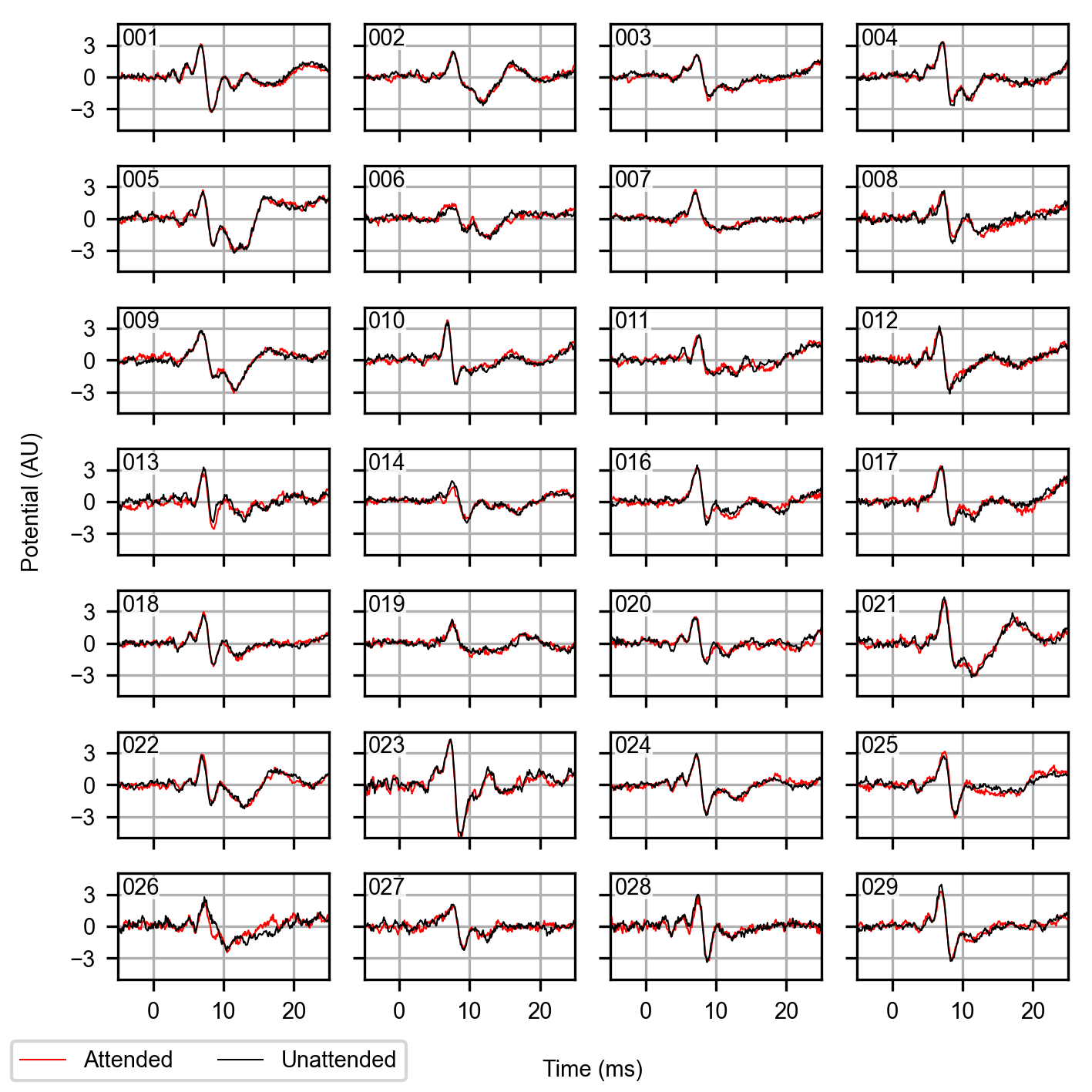

Supplement: S2 Fig — The ABRs to attended (red) and unattended (black) speech are shown for all subjects. Grand averages shown in Fig 1b. (TIF) [file pbio.3003407.s002.tif]

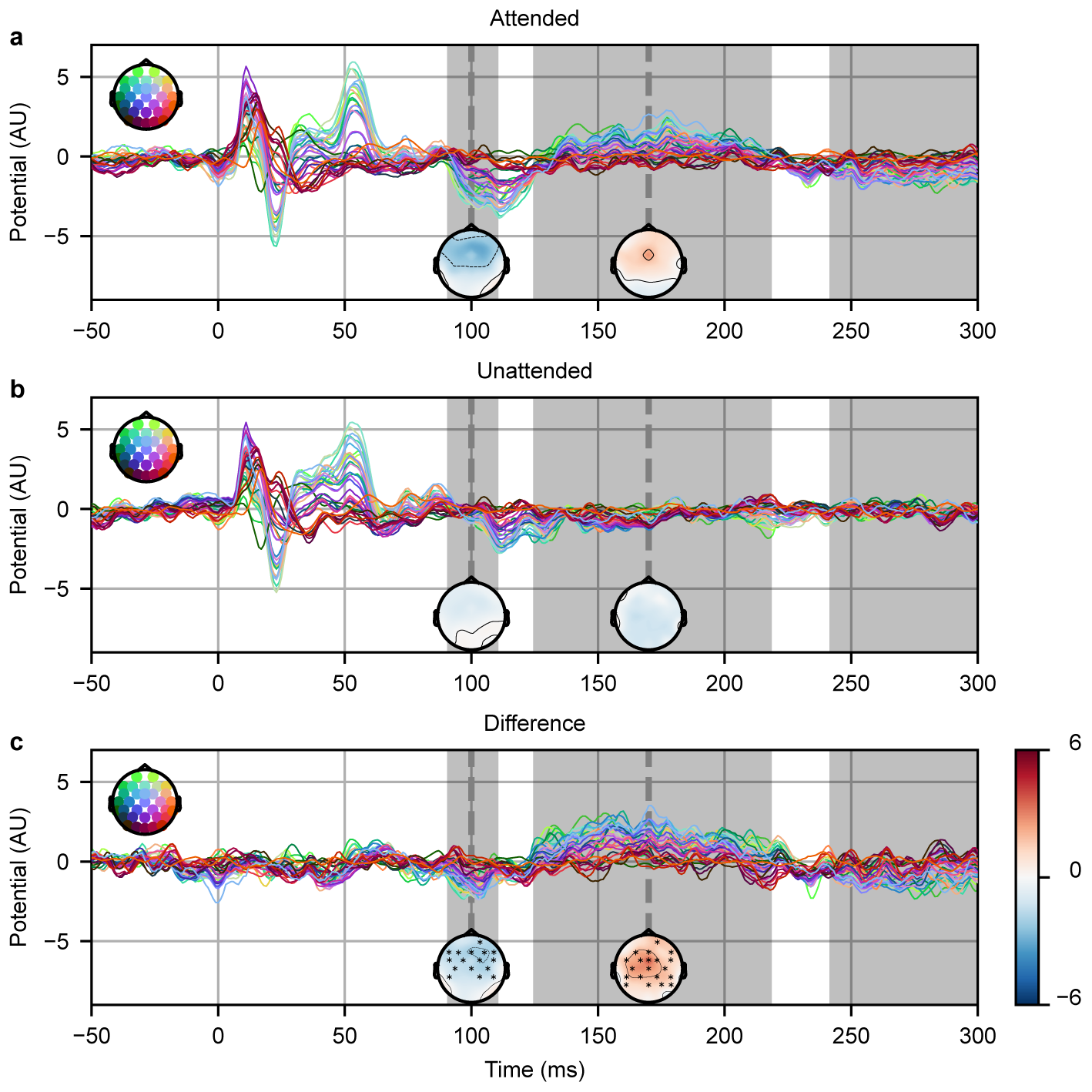

Supplement: S3 Fig — Cortical responses to attended, a, and unattended, b, stimuli. Each electrode is represented by a different color trace (key in upper left). Significant differences were observed in the 91–110, 125–218, and 242–300 ms regions (shaded on the time-series plots), as determined through paired, two-tailed spatiotemporal clustering methods (p < 0.05 for at least one electrode in the interval). Scalp topographies are shown for selected time points of 100 and 170 ms, denoted by a vertical dashed line on the time series plots. c, The difference waveform and scalp topographies at the selected time points. Asterisks indicate the electrodes that were significantly different across conditions at that time. (TIF) [file pbio.3003407.s003.tif]

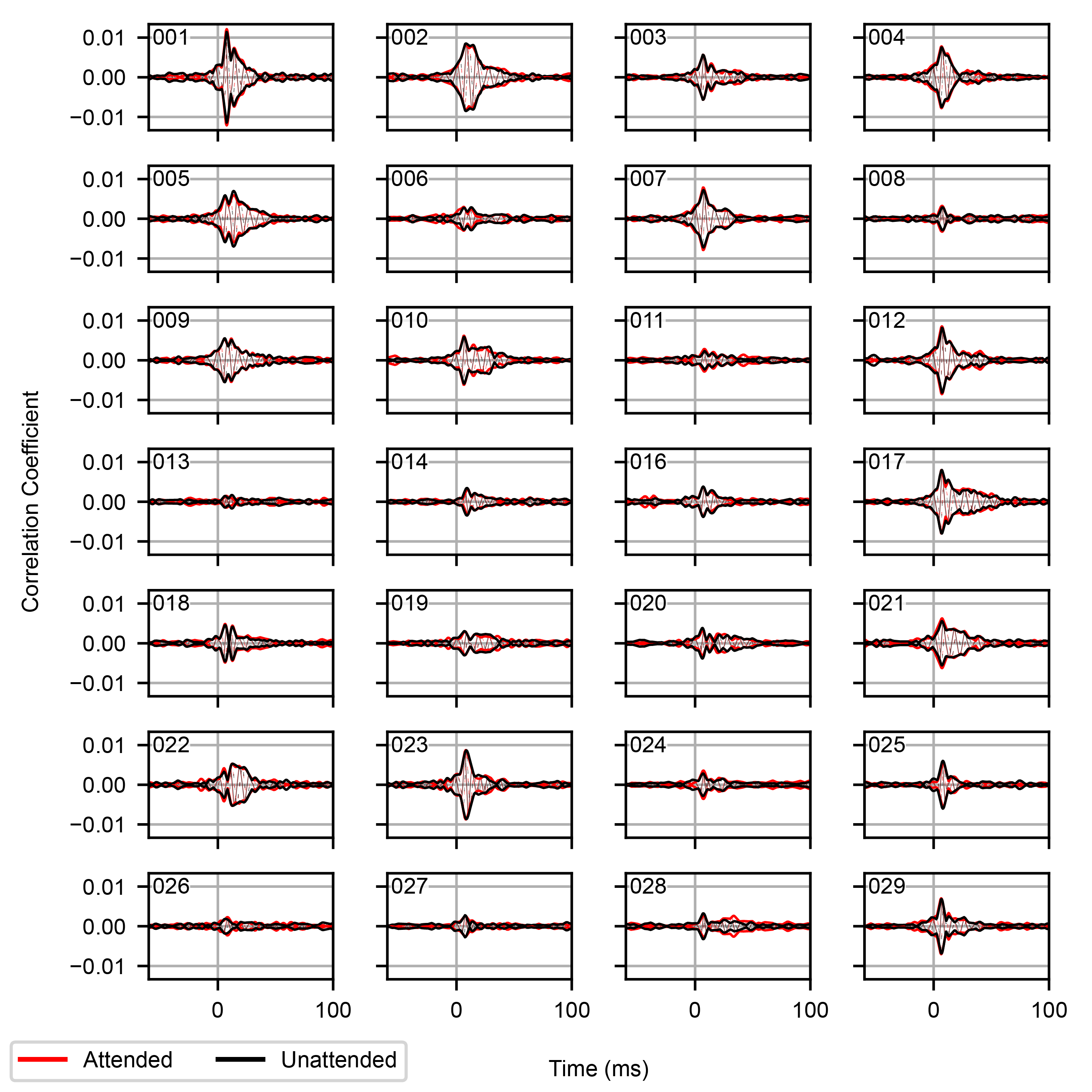

Supplement: S4 Fig — Responses to the attended (red) and unattended (black) speech are shown. Response peaks are shown in Fig 3. (TIF) [file pbio.3003407.s004.tif]

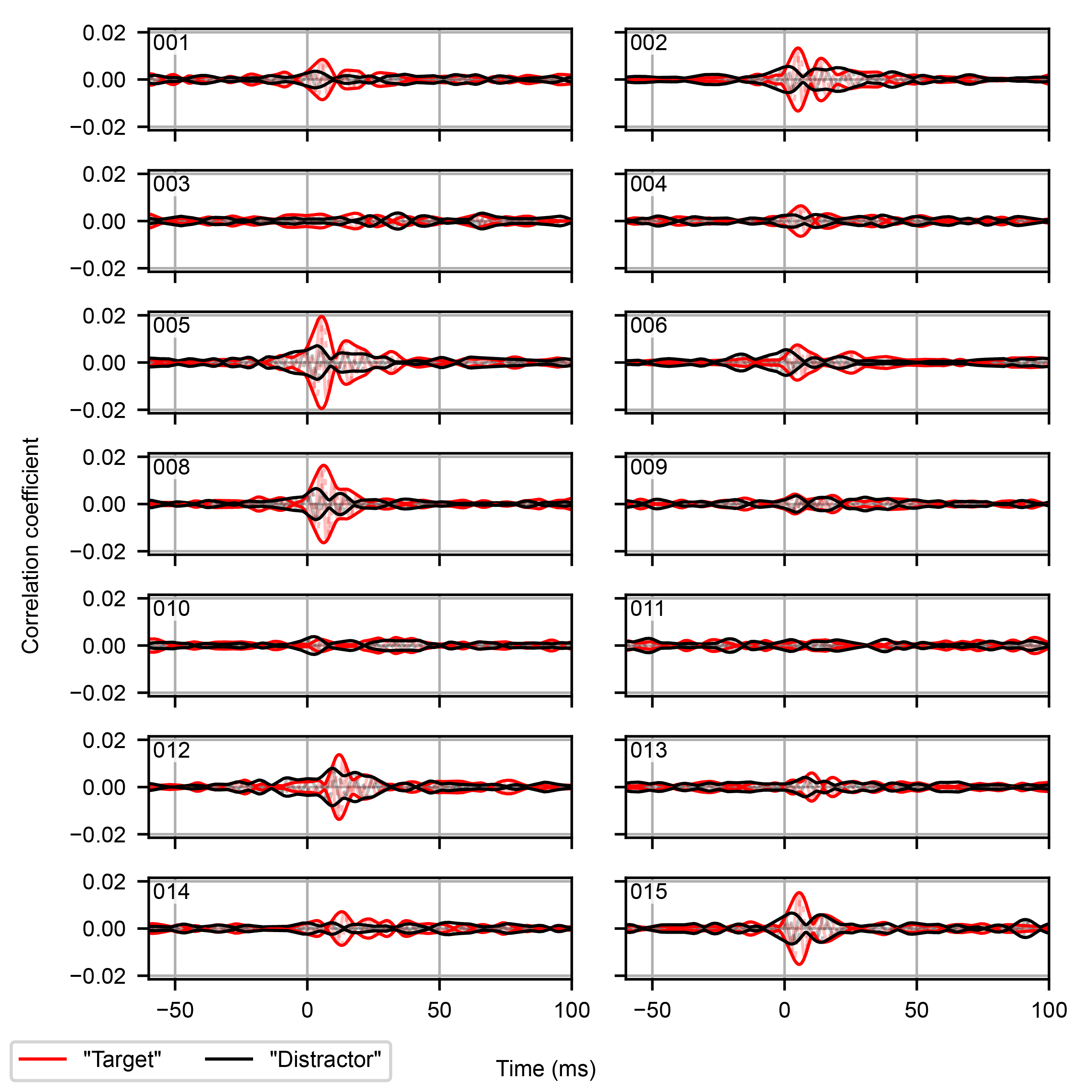

Supplement: S5 Fig — Responses to the “target” (red) and “distractor” (black) speech are shown. The “target” response can be clearly seen to be larger than the “distractor” response in several subjects, despite the task being passive. Response peaks are shown in Fig 4. (TIF) [file pbio.3003407.s005.tif]

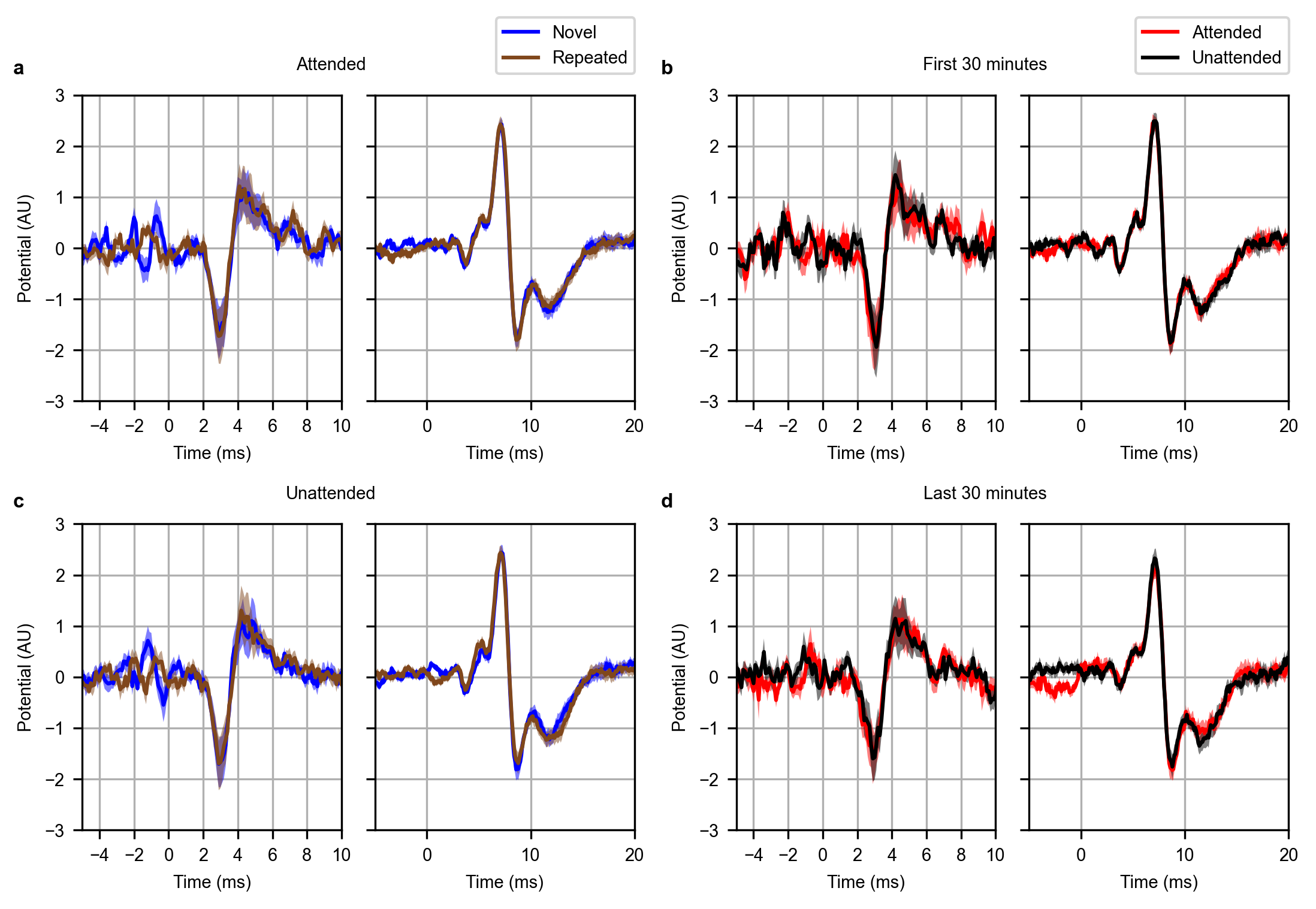

Supplement: S6 Fig — In order to ensure counterbalancing, all stimuli were presented exactly twice—once attended, and once unattended (order counterbalanced). Neither responses to attended, a, nor unattended, c, stimuli are impacted by whether the stimulus was novel or repeated. To address the possibility of the 2-hour experiment time impacting results (e.g., through waning interest), we analyzed separately the first 30, b, and last 30 min, d. There was no effect of attention at any time during the experiment. (TIF) [file pbio.3003407.s006.tif]

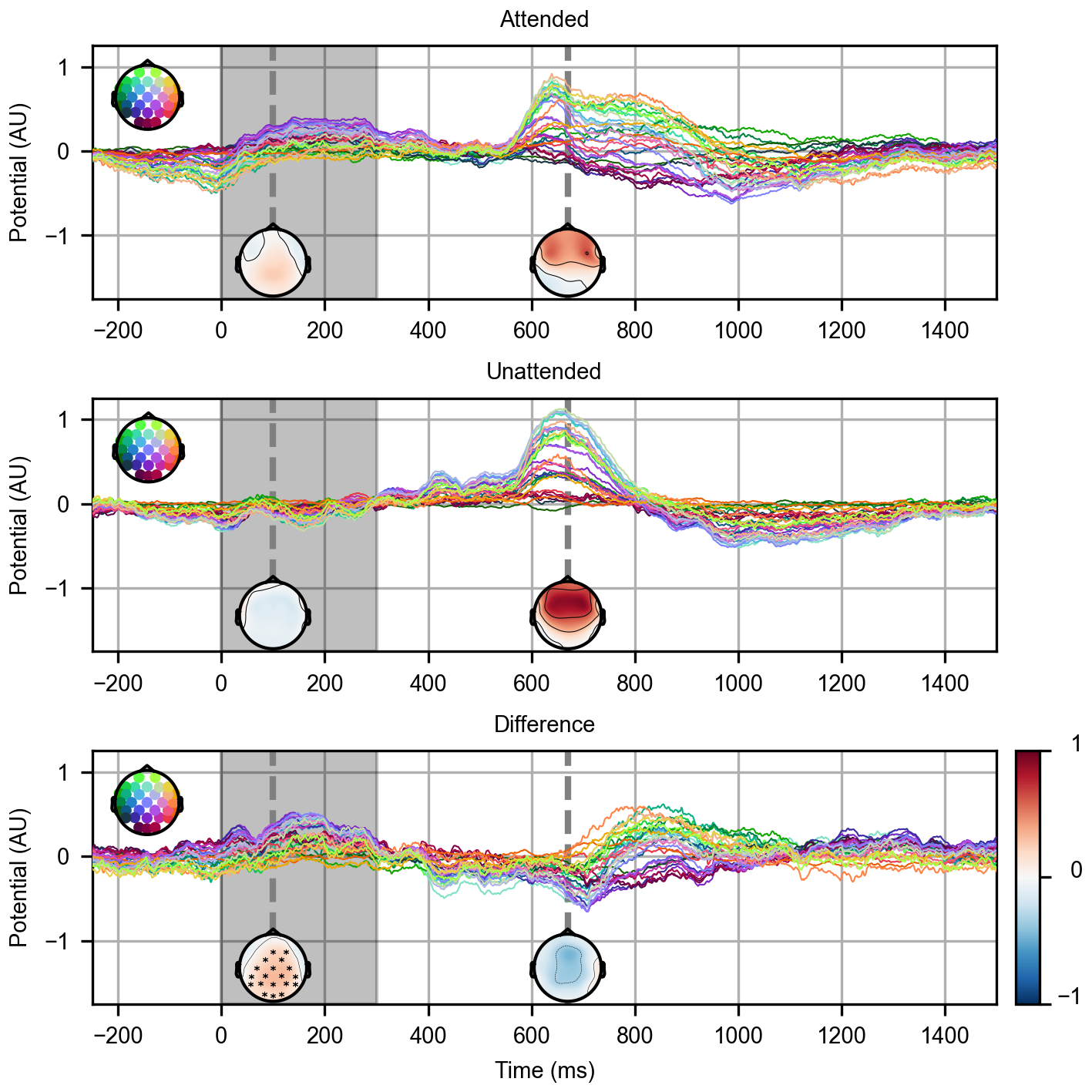

Supplement: S7 Fig — Cortical event-related potentials to the offsets in the a, attended and b, unattended speech streams. Offsets of speech stimuli occurred at zero on the time axis, and onset of the following speech occurred within the 490–500 ms time window. Each electrode is represented by a different color trace (key in upper left). Significant differences were determined through paired, two-tailed spatiotemporal clustering methods (p < 0.05 for at least one electrode in the shaded region). Scalp topographies are shown for selected time points of 100 and 670 ms, denoted by a vertical dashed line on the time series plots. c, The difference waveform and scalp topographies. Asterisks indicate the electrodes that were significantly different at the selected time point. (TIF) [file pbio.3003407.s007.tif]

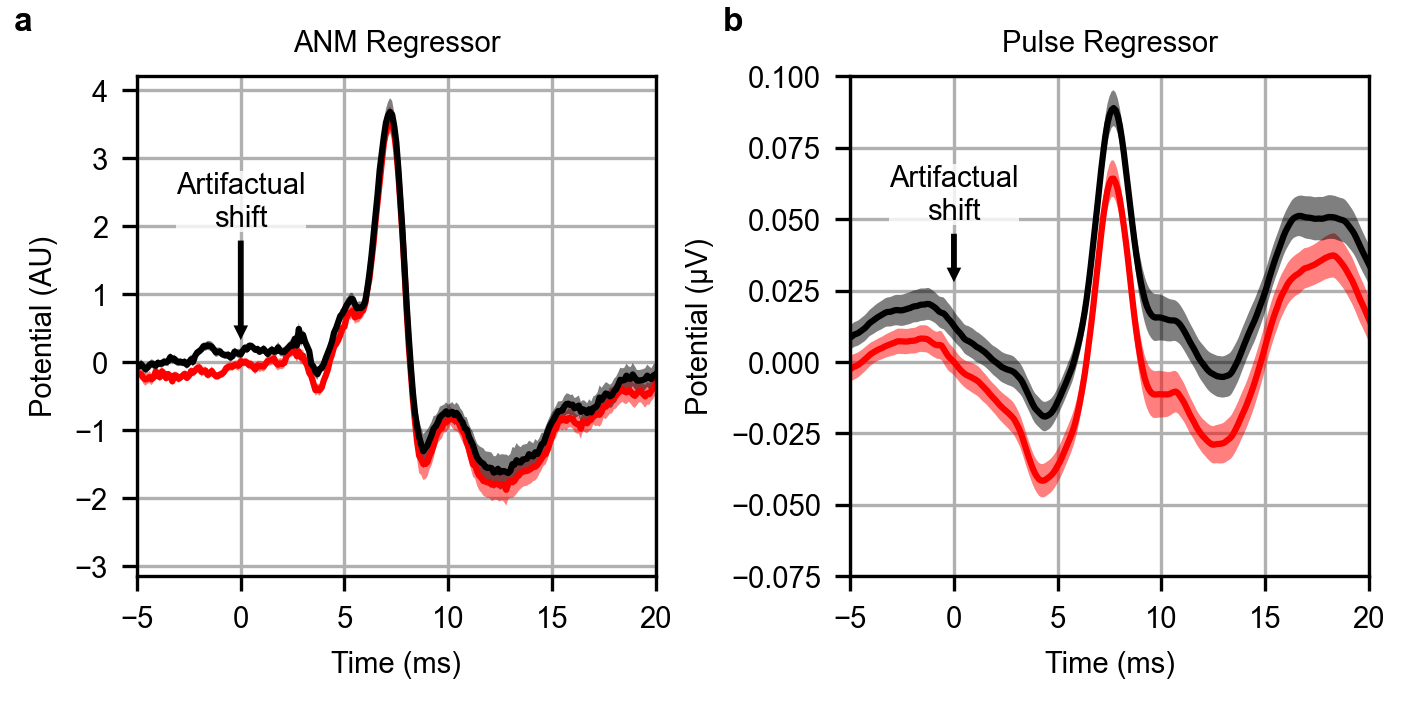

Supplement: S8 Fig — Without eliminating attention-dependent offset responses during the silent pauses (Fig 6), a spurious shift in the TRFs resulted. The shift wasslow and began well before 0 ms latency. It also depended on the specific TRF regressor used, and while present with the ANM regressor, a, it was even more pronounced when regressing against glottal pulses [39], b. Not eliminating the shift could have led to complications when interpreting the results. (TIF) [file pbio.3003407.s008.tif]
