# Supplementary material for: The auditory brainstem response to natural speech is not affected by selective attention
Source: PLoS Biol. 2025 Oct 6;23(10):e3003407. doi: 10.1371/journal.pbio.3003407 (PMC12500158; doi:10.1371/journal.pbio.3003407)
Supplement: Attachment — Submitted filename: Response to reviewers.docx [file pbio.3003407.s011.docx]

**Editor:** As you will see below, the reviewers think that the study your is overall well executed and provides important insights. However, in addition to the comments regarding the lack of methodological details and required textual revisions, for example regarding the length of the experiment and the impact of stimulus repetition, there is also a control experiment that will be required to address the reviewers' concerns, mentioned by Reviewer 2.

Reading the cross-comments, R2 agrees mostly with R1 and reiterates that the requested controls are necessary to support the claims in his opinion.

Given the extent of revision needed, we cannot make a decision about publication until we have seen the revised manuscript and your response to the reviewers' comments. Your revised manuscript is likely to be sent for further evaluation by all or a subset of the reviewers.

We thank the editor and reviewers for their valuable feedback. We have incorporated several controls and provided clarifications as described, strengthening this study. The title of the manuscript has also been updated. We have additionally corrected a bug in the analysis script for experiment 1 that was discovered while implementing these changes. This has slightly altered figures 1 and 2, as well as the values of the statistics, but the conclusions are the same. Please see detailed responses to the points raised by each reviewer in red text below. We have also uploaded a redlined version of the revised manuscript that highlights major changes.

**Reviewer #1:** The manuscript by Stoll et al. is an attempt to address conflicting results about whether or not subcortical auditory responses are modulated by selective attention to speech. Data were recorded in three experiments, where two concurrent audiobooks were presented to listeners, and they either had to pay attention to one of them (Experiments 1&2) or ignore them and engage in another task of their choice (Experiment 3). They utilize a unique recording setup to obtain neural responses from the auditory nerve (CAP), brainstem (ABR), and cortex (traditional scalp-EEG), which allowed them to separate the different levels of response.
Counter to results reported in previous studies, and primarily by Forte et al., they authors find no evidence for modulation of subcortical responses by selective attention, but replicate the well-established findings of cortical modulations. Moreover, they use the stimuli from the Forte et al. study in a passive-listening paradigm to claim that previous results may be related to stimulus-specific effect rather than selective attention.
I found this paper to be quite interesting and well executed, and I appreciate the methodological rigor for demonstrating the reliability of a "null" result. I believe that this work makes an important contribution in furthering this debate, and - while it may not be conclusive - provides compelling and important empirical evidence against selective-attention effects at the brainstem level (at least when listening to 2-hour long speech!).
I do, however, have several questions and missing details that I would appreciate if the authors could clarify:

1.  The stimulus-presentation scheme is not clear to me. Authors write that "The stories were ordered such that subjects could follow along, with each stimulus played twice (once attended, once ignored) so they would not miss sections of the story. At the end of each trial, subjects were asked two comprehension questions to keep them engaged in the task".
But many details are missing: Did they switch their attention between the male/female storied on each trial? Were the repetitions consecutive or were all the stimuli repeated in the 2nd half of the experiment?

We have added a table to the manuscript to more clearly detail the trial order (line 62, and copied here). Through this design, all stimuli were balanced such that all audio segments were presented twice (once attended and once unattended), equal amounts of male and female speech were presented while attended and unattended, and equal amounts of novel and repeated speech were heard and attended for each speaker. There were necessarily attention switches, but they were minimized within the constraints above so that they only occurred every fourth trial in the period between presentations. Please see below for the table:

Table 1: Stimulus presentation structure.

| Trial Number | 1 | 2 | 3 | 4 | 5 | 6 | 7 | 8 | 9 | 10 | … | 116 | 117 | 118 | 119 | 120 |
| --- | --- | --- | --- | --- | --- | --- | --- | --- | --- | --- | --- | --- | --- | --- | --- | --- |
| Story Segment | 1 | 2 | 1 | 2 | 3 | 4 | 3 | 4 | 5 | 6 | … | 58 | 59 | 60 | 59 | 60 |
| Attended Talker | F | F | M | M | M | M | F | F | F | F | … | M | M | M | F | F |

2. And on a more methodological level - is it possible that repeating the same content twice could affect speech tracking? One could imagine that hearing a story that I had previously listened to might be more distracting than an entirely new story.. were any control-analysis conducted to ensure this did not have an effect?

We have added a control analysis in Supplementary Fig. 6a,c to confirm there were no differences between the novel and repeated stimuli. With the trial order we used, the second of the repeated segments was either attended or unattended with equal proportions (see table above). Additionally, trials were never repeated immediately—there were always at least 2 minutes between repetitions.

>> the experiment was extremely long (over 2 hours!). I would expect attention to deteriorate over time or just general effects of fatigue. Can you comment on this? Did the behavioral performance change over time? This seems critical to me, since selective-attention is instructed here, but listeners may or may not have complied with this instruction, especially in such a long experiment. So. verifying that they were indeed paying attention as required is critical for interpreting the lack of selective-attention effects at the subcortical level.

We felt the experiment had to be long due to the low SNR of subcortical responses, which is lower with continuous stimuli and made worse by the addition of a second talker. Aside from encouraging breaks, the only thing that really helped with preventing exhaustion in the experiment was the books having interesting stories (most subjects had not read/heard them before, and several said they were going to finish them after the experiment).

We have added a second supplementary analysis (Supplementary Fig. 6b,d) examining the first 30 minutes and last 30 minutes of the experiment. While the responses from the last 30 minutes were slightly smaller overall, they were not significantly different from the responses from the first 30 minutes of the experiment. More importantly, when examining the first 30 minutes, the attended and unattended responses were still not different.

Thanks for these suggestions—we’re sure other readers will have some of the same questions.

3. Experiment 3: While I greatly appreciate the rationale of using the stimuli used by Forte et al. in a passive-listening design, in order to assess whether previously reported effects are potentially due to stimulus-specific features. While the distribution of single-subject data is nice to see, I am not fully convinced of this alternative explanation for several reasons:
a)      Results of passive-listening paradigms are very difficult to interpret, especially if they are long, since it's hard to know what participants are actually doing/paying attention to. It is quite possible that for at least some of the time, they listened to the speech, and that one stimulus 'grabbed' their attention more consistently. Do the authors have any insight into what participants actually did during this time? It seems unlikely to me that they were actively engaged in another task 100% of the time…

We carefully considered this possibility when planning the passive experiment, and designed it to ensure that it would not create a confound:

1. Story segments were presented in totally randomized order.
2. Each segment, when it came up in the order, was repeated four times in a row. Even if one story grabbed the subjects’ attention the first time, it would be unlikely to do so on the second, third, and fourth presentations.
3. Subjects were not given any information about what the stories would be, or even that there were two different stories from each narrator being presented. With the segments presented out of order, it would be hard for them to even ascertain that fact, and they would have to determine at the beginning of each segment which stories were being presented based on context.
4. The large majority of subjects would have had to agree on which two stories were worth attending, despite the above.
5. Finally, subjects brought in a book to read or chose videos from the internet to watch—they would not (perhaps even could not) have chosen something *less* engaging than these experimental stimuli.

Keep in mind also the strong evidence from the other two experiments that engagement with one story does not result in larger responses, even when using the analyses from Forte et al. (see Figure 4 in the manuscript).

We have added this reasoning to the Discussion section (lines 335-350)

b)      I wonder why the authors did not use these materials in a selective-attention paradigm as well - if they were able to show that similar responses are obtained during passive listening and "active/selective" listening, then perhaps this might be more convincing.

Forte et al. did the selective attention task, and our passive results matched theirs quite closely—the same two stories (the target male and target female) had larger responses than the other two stories. We didn’t see the need to repeat their experiment, given the passive results already matched. The design would have also been tricky, using the same stimuli as both passive and actively attended at different stages of the experiment.

c)      I also wonder why the authors chose not to show the waveforms of the neural responses obtained in the passive-listening experiment, which offer more direct comparisons to Experiments 1&2 and to prior work.

Individual waveforms are now shown in Supplemental Fig. 5. We do not show a grand average waveform because the ‘correct’ method of averaging (before/after envelope calculation) is not obvious, and was also not done in Forte et al. (they show an example subject instead), so it would provide no useful comparison.

d)      The authors claim that previous results may be due to "unbalanced" presentation of target and distractor speech (i.e., that they were not repeated), however I wonder if they have a hypothesis as to what speech-features might be driving the preference for one speech-stimulus over the other in their passive-listening conditions. This would be important for testing this possibility or controlling for acoustic differences in future studies (especially since repeating the same stimuli also has substantial drawbacks, as noted above).

It’s really tough to say what drives differences in responses. Extended Data Figure 3-1 here (https://www.eneuro.org/content/12/4/ENEURO.0561-24.2025) shows the responses to five different audiobooks spoken by male narrators with similar F0 and processed to have matching overall spectra (and thus also overall energies). Despite that, two of the stories (Peter Pan and Time Machine) produced quite smaller responses than the other three. This unpredictability underscores the need for careful counterbalancing of stimuli when doing experiments like this. This is now mentioned in lines 332-334.

4. For completion, it would be helpful if the authors presented the full results for Experiment 2 as well (CAP, ABR and scalp-EEG results).

The CAP does not exist for that experiment, since it was collected prior to development of the eardrum electrode. We have added the cortical results as Supplemental Fig. 3.

5. The "control" analysis for offset responses described briefly in the methods and in Extended Data Fig 1 sounds potentially important, but was not entirely clear to me. Could the authors please elaborate on this? One thing I found particularly confusing is that silences are not expected to be synchronized across attended and unattended speech, nor do they have constant durations (of 490-500ms). So, how should we interpret the ERPs shown here?

It's true that these silences were not synchronized across talkers. While their durations varied, those variations were relatively small. Essentially what happens is that these offset responses are much larger than the ongoing activity, and the regressor is zero-valued during the silence. As a result, for the first 500 ms or so of regressor after the pause, the offset response is computed as resulting acausally from the later regressor, and is smeared out since there is no part of the offset that is aligned with the regressor’s specific peaks. This negative-lag response masquerades as the brain “predicting” the attended speech, or somehow preparing for it. This would be a very exciting result if it weren’t artifactual.

Also, it would be nice what "artifact" the authors were trying to correct for in the TRF by zeroing the EEG during silences. Perhaps other studies suffer from a similar "problem"?

We have added Supplemental Fig. 7 to show this artifact. Whether other studies have encountered this problem is something we have also wondered about. Many other groups high-pass filter more aggressively than we do and/or baseline-correct responses, both of which could hide this issue. But it certainly will be important to consider for future studies that may report acausal effects in continuous stimulus designs.

**Reviewer #2:** This study investigates whether attention modulates subcortical responses using EEG and an eardrum electrode. While the methodology is rigorous, the framing of the findings is too general. For example, the title and the framing of the paper suggest there is no attentional modulation in subcortical areas, but this conclusion may depend on the type of stimulus used, the recording technique, and behavioral demands. These should be made more clear by making the claims more specific, than what is said in the title currently.

Prior studies (e.g., Slee & David, 2015) have reported subcortical attentional effects with simpler stimuli. One possibility is that attentional modulation emerges at different stages depending on stimulus complexity—when auditory features are easily separable, modulation could occur subcortically, but in complex scenes like multitalker environments, cortical processing may be required first. The study does not establish "when and where" attention acts, but rather what is observable with EEG. This distinction needs to be clearer.

We have updated the title of the paper to “The auditory brainstem response to natural speech is not affected by selective attention.” We discuss the limitations of the study (and EEG in particular) in the “Caveats and conclusions” section of the Discussion.

The paper would also benefit from a stronger connection to theoretical models of brain function. If subcortical attention effects are absent, does this support a strictly feedforward model of auditory selection? What does this imply about how the brain processes complex acoustic scenes?

This was definitely missing from the manuscript. We’ve added two paragraphs to the discussion that touch on this and some related issues (lines 228–246). Thanks for the suggestion. Here are the paragraphs, for convenience:

Efferent pathways are pervasive throughout the auditory system and influence subcortical sound encoding in several ways^53–55^, with additional functions surely still to be discovered. At the lowest level, the periphery receives projections from the superior olivary complex via the olivocochlear bundle (OCB). The OCB has been has been shown to play a role in gain control^56^ and protection against high sound levels^57^, with several other possible functions suggested as well^53^. Rapid and frequency-specific filtering would be needed to selectively enhance the AN response to one sound in a mixture, but prior research has suggested that effects of the medial OCB may be both too slow to keep up with speech's dynamic structure and too broadband to be selective^56^. Consistent with this, we found no effect of selective attention in the auditory nerve (assessed with the CAP) in our experiment.

A selective attention effect could arise later in the subcortical pathway, such as the inferior colliculus, which is the recipient of extensive efferent corticocollicular projections^58^. Like the olivocochlear bundle, several roles for these efferents have been suggested^59,60^, with selective attention among them. Yet here as in the periphery, we find evidence against selective attention effects in the inferior colliculus or other midbrain nuclei, indicated by perfectly overlapping ABR waveforms and no wave V differences. Therefore, our results indicate that efferents with subcortical targets are not selectively enhancing the encoding of individual speech streams in a mixture, whether they are superimposed or presented to separate ears. We note, however, that these findings should not be construed as indicating that subcortical efferents don’t affect subcortical stimulus encoding in other important ways. Additionally, our results also do not indicate that no efferent projections are involved in selective attention—it seems a near certainty that top-down attention relies on at least intracortical efferent projections, but that is beyond the scope of what this study was designed to investigate.

A critical missing control is in Experiment 3, where the authors argue that prior studies mistakenly attributed acoustic differences to attentional effects. However, their passive listening design does not confirm that subjects were truly disengaged. Without behavioral verification, it remains possible that subjects were involuntarily engaged with one stream over another, possibly with the more salient/engaging story. A stronger test would be an internal control within their own dataset: splitting attended and ignored stimuli into non-overlapping time segments (e.g., first half attended, second half ignored). If their claim is correct, this artificial separation should create a spurious "attention effect" purely due to acoustic differences. Without this test, their critique of prior work remains speculative. Conducting this additional control would provide a stronger argument that prior findings were driven by acoustic confounds rather than genuine attentional modulation.

We ran the control experiment, and we did see the reviewer’s suggested “spurious ‘attention effect’” (see below—filled bars are generally higher than empty bars). We caution, however, that the differences there may be more driven by a slight overall decrease in response size at the end of the experiment compared to the beginning (this is why we include it here but not in the paper).

The stronger case against “accidental” engagement with the target stimuli is made in our response to reviewer 1—it just isn’t a plausible explanation given several aspects of the experimental design. Please see that response or the additions to the Discussion section (lines 335–350) for a fuller description of how our design prevents the issue.

Minor points:
- Justification of dichotic stimulus presentation requires more citations. (e.g. this sentence: . If the sounds were spatially separated, a much simpler scheme could be employed wherein the better ear was favored)

We’re not sure what citation we could add here—the point we were trying to make is akin to the fact that it is much easier to turn the volume up on just one person if they’re using separate microphones (i.e., dichotic) rather than both talking into the same one (diotic). The former is trivial while the latter would require some sort of computational speech separation algorithm.

To make that clearer we have changed “spatially separated” to “different in each ear,” which is more accurate anyway.

- In Figure 2: Would a difference plot (attend minus unattended) or an alternative visualization help clarify the effects, particularly for early vs. late responses?

We have updated the plot to include the difference waveform.

**Reviewer #3:** This study examines how selective attention influences sound encoding along the auditory pathway. Using a novel experimental setup, Stoll et al. simultaneously record neural responses from multiple levels of the auditory system. While prior research suggested that attention might modulate subcortical processing, their findings provide strong evidence that selective attention does not impact sound encoding in the auditory periphery or brainstem, with attentional effects first emerging in the cortex.

The study is well-executed, incorporating various control experiments (e.g., diotic vs. dichotic conditions, data reanalysis using methods from Forte et al.) to account for potential null findings. Additionally, the manuscript offers a plausible explanation for at least one previous report of attentional modulation in subcortical processing.

I find this to be an excellent piece of work that merits publication. However, I have one consideration: the authors chose to repeat each stimulus twice, once attended and once unattended conditions. They have a good reason for making this choice, as changing stimuli between conditions introduces acoustic differences that might confound attention effects (as the authors suggest happened in the study by Forte et al.). However, the impact of stimulus repetition should not be overlooked—not only in terms of changes in neural responses to first vs second stimulus presentation, but also regarding cognitive factors such as task engagement and cognitive load. Could the repetition of stimuli have reduced the likelihood of detecting an attentional effect?

We have added a control analysis in Supplementary Fig. 6 to confirm there were no differences between the novel and repeated stimuli. With the trial order we used, the second of the repeated segments was either attended or unattended with equal proportions (see new Table 1 in the paper). Additionally, trials were never repeated immediately—there was always at least 2 minutes between repetitions.
